# Barriers and Facilitators to Caring for People Presenting With Suicidal Distress in Rural Emergency Departments: A Qualitative Study

**DOI:** 10.1111/inm.70269

**Published:** 2026-05-15

**Authors:** Corina Modderman, Maria Veresova, Rachael McAleer, Lisa Hanson, Laura Hemming, Phil Maude, Evelien Spelten

**Affiliations:** ^1^ Violet Vines Marshman Centre for Rural Health Research. La Trobe Rural Health School – La Trobe University Bendigo Campus Flora Hill Victoria Australia

**Keywords:** emergency department, emergency service, hospital, mental health services, practice, rural health, suicidal thoughts and behaviour, suicide, suicide prevention

## Abstract

This qualitative study explored rural emergency department (ED) staff experiences of caring for people presenting with suicidal thoughts and behaviours and examined perceived barriers and facilitators to providing best care. Semi‐structured individual interviews and focus groups were conducted with 18 ED staff across two rural hospitals in Victoria, Australia. Participants included nurses (*n* = 15), mental health clinicians (*n* = 2) and a social worker (*n* = 1). Data were analysed using reflexive thematic analysis following Braun and Clarke's approach, and reporting was guided by the COREQ checklist. Four overarching themes were identified: the central role of people and relationships in shaping care; inadequate resourcing of rural EDs and community services to respond to suicidal presentations; current ED processes that hinder timely and responsive mental health care; and insufficient education and training regarding suicidal presentations. Staff described strong commitment and compassion in their care, alongside distress arising from long wait times, unsuitable ED environments, limited specialist support and gaps in follow‐up care. Structural and systemic constraints were perceived to compromise both consumer safety and staff wellbeing. The findings highlight the challenges rural ED staff face in delivering timely, person‐centred care to people in suicidal distress and demonstrate the need to strengthen environments, workforce capacity, care pathways and training to improve the quality and safety of care in rural ED settings.

## 1 Introduction

Suicide is a major public health concern in Australia. In 2023, there were 3214 deaths by suicide recorded, with an age‐standardised rate of 11.8 per 100 000 people (Australian Bureau of Statistics 2024), highlighting the ongoing need for evidence‐informed and compassionate care across acute settings. Emergency departments (EDs) are a frequent point of contact for people in suicidal distress and those who have self‐harmed, including children and young people (Hudson and Randall 2026). Mental health‐related ED presentations increased in 2023–2024, with only 60% of consumers seen on time against the triage benchmark, which demonstrated capacity and timeliness issues (Australian Institute of Health and Welfare (AIHW) 2025).

Research on consumer perspectives of visiting the ED consistently describes stigmatising attitudes, long wait times, insufficient emotional support and the unsuitability of the ED environment for psychological distress (Brousseau‐Paradis et al. 2024; Navas et al. 2022; Shand et al. 2018; Liddicoat 2019). Similarly, young people share negative experiences of ED care for suicide‐related presentations (Freeman et al. 2022; Virk et al. 2022). Following a hospital presentation for self‐harm, the risk of suicide is highest in the first month and year after discharge, demonstrating the importance of timely and integrated care follow‐up (Geulayov et al. 2019). A recent study reported that only 41% of Australians presenting in suicidal distress receive follow‐up care (Spittal et al. 2024). The lack of appropriate follow‐up can reduce willingness to seek help in the future (Shand et al. 2018) and may increase future suicide risk (Hill et al. 2023; Veresova et al. 2024).

Rural, regional and remote locations in the Australian context add a further layer of complexity. The heightened suicide risk in rural communities is driven by a complex interplay of factors, including social isolation, stigma around mental illness and increased availability of lethal means (Fitzpatrick et al. 2021; Kennedy et al. 2020; Mohatt et al. 2021). Notably, suicide rates increase as population density decreases, with residents of very remote areas recording a rate of 21.0 deaths per 100 000 population, more than double the rate of 10.0 recorded for major city residents (AIHW 2024). Australian health‐workforce policy recognises gradations of remoteness on the Modified Monash Model (MMM) from MM1 (metropolitan) to MM7 (very remote); areas MM2‐MM7 are considered regional, rural and remote (referred to as rural from here on) and face persistent issues around access, staffing, and infrastructure challenges (Australian Government Department of Health and Aged Care 2025a). Furthermore, a significantly higher proportion of rural Australians compared to urban Australians report not having a general practitioner or a specialist nearby as a barrier to accessing healthcare (AIHW 2018).

EDs often become the primary point of care for individuals presenting with suicidal distress, even more so in regions where mental health services are scarce and specialist support is limited (Grover et al. 2023; McCarthy et al. 2024; Rheinberger et al. 2022). The Psychiatry Supply and Demand Compendium Report (Australian Government Department of Health and Aged Care 2025b) highlights that mental health professionals are concentrated in major cities, leaving significant service gap in rural areas. The lack of a skilled workforce reduces the capacity of health systems to respond effectively to individuals experiencing suicidal thought and behaviour. Australia is projected to have a significant gap in its psychiatry workforce over the next 25 years, with an estimated undersupply of 20.7% by 2048, highlighting the urgency of the issue (Australian Government Department of Health and Aged Care 2025b). There is an uneven distribution of the specialised mental health workforce, with fewer workers as distance from metropolitan centres increases (AIHW 2024), with significant spatial inequality in the availability of rural health professionals compared with urban areas.

The rural context adds to the general barriers experienced by EDs in delivering optimal care, potentially highlighting different priorities and challenges. Presently, research focusing on suicide prevention in rural EDs in Australia is limited. Existing studies have found that suicidal ideation or self‐harm are the most common mental health presentations in rural EDs (Pawaskar et al. 2022) and almost a third of these were consumers re‐presenting for a suicidal crisis, emphasising the importance of adequate follow‐up support (MacDermott et al. 2023). However, ED staff highlight the lack of available community mental health services in rural areas (Pawaskar et al. 2022), leading consumers to feel that their needs were not fully met and re‐presenting to the ED.

The Royal Commission into Victoria's Mental Health System (Victorian State Government 2021) has identified a need to improve ED capacity to respond to mental health crises across the state. Further, it recommended that each region (including rural locations) should have EDs that are equipped for mental health‐related presentations (Victorian State Government 2021). However, given the barriers, ED care is currently sub‐optimal. Studies with rural ED staff suggest that existing barriers may be further exacerbated by the rural location, such as inefficient patient transport leading to longer wait times, shortage of specialist mental health staff and limited opportunities for ED clinicians to receive training on suicidal presentations (Pawaskar et al. 2022). As such, there is a need for an in‐depth examination of how rurality shapes ED service provision, and how care can be improved for suicidal consumers (MacDermott et al. 2023).

To address the limited evidence on rural ED responses to suicidal distress, this study aimed to examine ED staff experiences of caring for people presenting with suicidal thoughts and behaviours, and to identify the key barriers and facilitators influencing care delivery.

## 2 Methods

### 2.1 Study Design

A qualitative approach was employed, involving individual interviews and focus groups with emergency department (ED) staff across two rural hospitals in Victoria, Australia. The study was underpinned by an interpretivist epistemological approach, recognising that participants' accounts reflect socially and contextually constructed meaning (Crotty 1998). The Consolidated Criteria for Reporting Qualitative Research (COREQ) checklist (Tong et al. 2007) guided both study design and reporting, informing decisions about research team composition, sampling approach, data collection methods and analytic procedure. Ethics approval was obtained from La Trobe University Human Ethics Committee (Approval number: HEC25032).

### 2.2 Participant Selection and Recruitment

Recruitment was facilitated by hospital site managers, who distributed study information via email to all ED staff. A purposive convenience sampling approach was used. All staff who expressed interest in participating were enrolled, resulting in a final sample of 18 participants. Participants were invited to participate by completing an online consent form and a brief demographic questionnaire.

### 2.3 Setting and Data Collection

The research team contacted participants to schedule an individual interview or focus group according to their preference. Data collection sessions were conducted either online or in‐person at the hospital site. Six individual interviews and three focus group discussions (each with four participants) were conducted, following established methods for qualitative interviewing (Baillie 2019; Guest et al. 2020) and focus group research (Roald et al. 2025). Individual interviews ranged from 25 to 88 min (median 72 min); focus group discussions ranged from 28 to 42 min (median 41 min). Each session was audio or video recorded. Participants received a $100 gift card as reimbursement for their contribution.

Data were collected across two rural hospitals in Victoria, Australia. The first, classified as MM2 on the Modified Monash Model, serves a catchment area of approximately 300 000 people and receives over 70 000 ED presentations annually. The second, classified as MM3, serves a population of approximately 150 000 people and receives over 40 000 ED presentations annually.

Interviews and focus groups followed a semi‐structured question schedule exploring perceived barriers and facilitators to supporting people in suicidal distress and collaborating with other service providers. Participants were asked about their experiences of working within the immediate ED team and the broader healthcare system.

### 2.4 Data Analysis

Data were analysed using Braun and Clarke's (2021) reflexive thematic analysis, an approach that positions researchers as active agents in the construction of themes rather than passive identifiers of pre‐existing patterns. Rather than cataloguing participant responses, this method involves working interpretively with the data, generating, developing and reviewing themes through an iterative process. Themes reflect the researchers' analytic choices and theoretical orientation, not simply the frequency with which ideas were mentioned. Interviews and focus groups were analysed together, with attention to both individual experience and shared or contested meanings arising through group discussion.

Authors MV and RM independently coded and analysed the data using NVivo software (QSR International Pty Ltd). An initial coding framework underwent refinement through an iterative cross‐checking process. Service managers with direct experience of rural ED mental health care were consulted throughout to review emerging themes and provide feedback on their resonance and clarity (Denzin and Lincoln 2011).

Reflexivity was central to the analytic process. All authors brought clinical and/or research experience in mental health and emergency care, which informed both the questions pursued during data collection and the interpretive lens applied during analysis. Reflexive discussions were held regularly throughout, and assumptions were critically examined as themes developed. For example, an early interpretation that framed wait times primarily as a systemic failure was challenged through team discussion, leading to greater attention to the interpersonal and emotional dimensions participants described. These discussions supported critical scrutiny of emerging interpretations and contributed to the credibility and rigour of the analysis.

### 2.5 Rigour and Trustworthiness

Credibility was further strengthened by including participants' own quotations, directly linking researchers' interpretations to practitioners' experiences and perspectives (Denzin and Lincoln 2008). To protect anonymity, pseudonyms were assigned and identifying details, including specific professional roles, were not reported. Credibility was also enhanced through careful contextualisation within the Australian rural ED landscape (Patton 2002). Rich contextual description supports informed judgements about the transferability of findings to other settings (Lincoln 2007).

## 3 Findings

To address the limited evidence on rural ED responses to suicidal distress, this study examined staff experiences of caring for people presenting with suicidal thoughts and behaviours and identified key barriers and facilitators influencing care delivery. Eighteen ED staff participated. Participants included nurses, mental health clinicians and a social worker, representing a range of ages and professional backgrounds. Their accounts generated four overarching themes: (1) relationships and interpersonal care; (2) environment and resources; (3) processes and delays; and (4) training and preparedness. Participant characteristics are presented in Table 1.

**TABLE 1:** Demographic characteristics of participants.

Category	*n* (18 total)
Gender	
Female	13
Male	5
Age	
18–24 years	1
25–34 years	8
35–44 years	3
45–54 years	6
Ethnicity	
Caucasian	15
Asian	2
Prefer not to say	1
Current role	
ED nurse	15
Mental health clinician^a^	2
Social worker	1
Years worked in ED	
Less than 1 year	1
1–5 years	8
6–10 years	3
11–20 years	4
More than 20 years	2

^a^ Mental health clinicians are located within the hospital and respond to mental health‐related ED presentations after they have been triaged.

### 3.1 The Central Role of People and Relationships in Shaping Care

The interpersonal aspect of providing mental health care in the ED was discussed in detail by the participating staff. Participants expressed a strong commitment to supporting consumers in suicidal distress, describing compassion and active listening as essential aspects of care. Offering human connection in moments of crisis was seen as core to their role:

Seeing the patient as a human being and trying to alleviate as much of that anxiety as possible. […] Making sure they have comfort and privacy as much as possible.—Peter

While participants felt competent at building rapport with consumers, some participants said that managing relationships with parents or carers who accompany the consumer to the ED could sometimes present challenges: “Some of the hardest conversations I have is with anxious family members” (Anita). This was deemed particularly concerning regarding confidentiality around young people under the age of 18. Alice, expressed challenges when supporting young consumers' families; while many parents wanted to help, there were limited structured opportunities for education or inclusion in decision‐making, especially if the young person wanted the details of their presentation to remain confidential:

The mental health workers, […] they do the mental health assessment in there, don't give the parents any feedback. Then [the young person is] sent home. […] And parents have to deal with it without knowing what's happened. […] You don't have to divulge confidential things that was discussed in the assessment, but just say, give them some guidance about how they can help their child.—Mia

Another interpersonal challenge that complicated the delivery of optimal care was when consumers act in an aggressive manner. William described it as “so detrimental to the way a department runs on that shift.” Participants explained that aggression interfered with the quality of care provided, and the safety of staff and other consumers:

It heightens fear in the department, which then means that other patients are not looked after as well. So, there's a big knock‐on effect from that one person who is aggressive, loud, vocal, maybe not physically threatening but got the potential to do that.—Jonnie

Physically, physiologically, you can't empathize with someone and develop a relationship with them if they're a risk to you.—Helen

### 3.2 Environment and Resources

Participants unanimously felt that the ED as a physical environment is not well‐suited for people in a suicidal or mental health crisis. The ED was described as a stimulating space that is not conducive to therapeutic engagement:

The environment itself is not really set up for peace and quiet and to feel supportive. It's a very clinical harsh environment. The lights are always on, there's always people rushing around.—Ricky

The shortage of private waiting areas or assessment rooms within EDs was also deemed a significant drawback:

We often don't have anywhere to put people who are crying. So unfortunately, when there is the wait period, they have to just be exposed to the public in their emotional state.—Alli

There's just simply nowhere really private to speak to them.—Alli

Furthermore, participants highlighted that there is often not enough mental health staff in the ED to provide these consumers with the specialised care they need:

It feels like there's only 1‐2 [mental health staff] working per shift. They're dealing with our presentations, and their case presentations, and every call on that mental health hotline. So, they're just inundated.—Ricky

There was strong support for a dedicated mental health area within ED that is more responsive to the needs of people who present for suicidal reasons:

A little section that is mental health specific that is managed and monitored by mental health trained staff, even if they're co‐trained, would be really beneficial.—Els

Participants made multiple suggestions for this area. Peter suggested having “less beeping” from medical devices, “comfy armchairs”, and “quiet interview rooms available” for assessing people. Mia spoke about having “a space for them to wait […], or at least some kind of a screen between them and the other patients in the waiting room”.

Participants described that when people presented for suicidal reasons to other community services, such as urgent care centres or general practitioners, they were usually referred to the ED, as Els stated, “All roads come to ED here”. However, participants perceived this to be concerning because for many people, a suicidal crisis is related to social, financial, or other stressors, which the ED is not equipped to resolve. The need for better community‐based options to manage suicidal presentations outside the ED setting was deemed crucial:

The end consequence is they turn to ED as their last resort, and pretty much we kind of wrap a bandage around them, put them back out again. […] They'll be seen in the community but then they'll come back in a few days, and no one's phoned them.—Anita

This issue was seen as particularly concerning for young people, since inpatient facilities for youth in rural areas are even more limited than for adults. Maya explained that “for an inpatient [admission], they have to travel 3 and a half hours to Melbourne, if there's something available”. If a place was not available, the young person would be discharged home.

### 3.3 Processes and Delays

Participants described current ED processes of caring for consumers in suicidal distress as falling short of delivering timely and appropriate care. They said that it is common for people to wait for a long time in the ED, and in sub‐optimal conditions, to see a mental health clinician:

We will often have [people with] suicidal ideation, particularly the ones that don't have a plan, they can wait for up to 10h to be seen by a mental health specialist. They're not often provided a bed while they're waiting. They're often in the waiting room.—Marcus

Extensive wait times often resulted in consumers leaving before their episode of care is complete, which created a moral dilemma for participants:

They've waited 3 and still haven't been seen, they've had enough. They've either changed their mind and they're like, “I don't really want to do – I'm not suicidal”. Then they want to go home, but then they would express those suicidal thoughts. So, then it's that duty of care, like, we can't let them go because if something happened then that would be on us.—Mimi

Participants felt that one factor contributing to long wait times was the requirement for consumers to be fully medically cleared or detoxified before receiving mental health care. This was perceived as a barrier to timely and comprehensive treatment:

Sometimes patients will be alert and talking to you, but their blood tests are a bit out of whack still. So, they're still under the care of the emergency doctors. But there's no reason that we need to delay their care by hours and hours and hours to make sure they're 100% good to go before they even get assessed by the mental health team.—Leah

Participants noted that the process, that does not allow for concurrent medical and mental health care, leads to consumers having to repeat themselves, which increases their psychological distress:

I often sympathise with them when they get frustrated that they find themselves telling their story to many different clinicians. They're having to repeat themselves.—Leah

Another way in which processes could be improved is by introducing a behaviour observation tool with numeric values to track consumers' mental state. Mia said that observational tools are widely used to monitor vital signs and physiological state for medical presentations, however, analogous tools were seldom used for mental health presentations. She felt that these tools with numeric values would aid nurses in appropriately responding to changes in consumers' mental wellbeing, including deciding when to escalate care:

We can say their behaviour is abnormal, but we can't track how abnormal or how far from their normal they have become. […] There's power in being able to say, […] “their observation has changed. They were scoring 0, now they're scoring 5. That's a big change”.—Mia

There was a clear desire for processes that match the urgency and complexity of suicidal and mental health crises, ensuring that consumers are supported through the full care journey.

### 3.4 Training and Preparedness

Participants emphasised that there was a lack of training on suicidal presentations throughout their formal education, as well as in professional development opportunities. Some felt underprepared when first starting in the ED and reported having to “learn on the job how to approach these people” through exposure and “trial and error” (Mimi), or by “[learning] off other nurses” (Helen). However, participants noted that the skills modelled by other nurses were not necessarily well‐informed or best practice:

If you were trained by someone who has misunderstood or is awkward themselves, they are less likely to encourage you to do that or to show you how to [talk about suicide] because they themselves struggle with it. So, if you weren't someone who that was privy to a sort of, for lack of a better word, braver triage nurse, you may never learn the language to do that with.—Alice

This lack of confidence and comfort with asking consumers about suicide was at times perceived to be detrimental to optimal care, impacting both the consumer and participants:

Sometimes [staff] will avoid asking what they might interpret as vulnerable questions because they don't want to upset the patient. Sometimes what they actually need is someone to ask direct questions so they get the care they need as fast as they need. So, it can be a difficult process for everybody.—Els

Participants largely felt that there was a mismatch between the medical care ED staff were trained to provide and the emotional support that these consumers needed:

We try to give them basic courtesy, empathy. We try to look after them and their basic needs, but we are not going to fix their mental health problems like we can fix someone with a broken arm. […] We're not trained to fix that.—Helen

Jonnie observed that some staff felt hesitant to provide mental health care because it is not “within their scope” as an ED nurse and they “haven't been specialised in that area”. Jonnie emphasised the need for a “collaborative approach between mental health and medical training” that is led by the “in‐house mental health team who we liaise with, who know the processes here”.

## 4 Discussion

This study addressed the limited evidence on how rural EDs respond to suicidal distress by examining participants experiences of caring for people presenting with suicidal thoughts and behaviours, and by identifying key barriers and facilitators influencing care delivery. Four themes were identified: (1) the central role of people and relationships in shaping care; (2) environment and resources; (3) processes and delays; and (4) training and preparedness. Together, these findings demonstrate that while ED staff are committed to providing compassionate, person‐centred care and working collaboratively with mental health clinicians, structural and systemic conditions in rural settings frequently hinder timely and safe responses.

The pattern described across multiple themes, in which people in suicidal distress are consistently redirected toward the ED regardless of where they first present, was captured directly by one participant that observed that “all roads come to ED here”. This captures the structural reality most participants described: that community services, urgent care centres, and general practitioners routinely refer people in crisis to the ED, even when the presenting concerns are social, financial or psychological rather than medical in nature. This dynamic reflects not a failure of individual services, but a systemic gap in the availability and resourcing of community‐based mental health care in rural areas, a pattern well documented in the broader literature (Pawaskar et al. 2022; Rheinberger et al. 2022).

Participants emphasised compassion and active listening as core to responding to suicidal distress, while also describing the interpersonal complexity of engaging family/carers and managing consumer aggression within busy EDs. These accounts echo consumer reports of insufficient emotional support and negative ED experiences for suicide‐related presentations, including young people (Freeman et al. 2022; Veresova et al. 2024). In the case of adolescent consumers, clarifying when and how to include parents/caregivers emerged as a practical need, consistent with research calling for structured family involvement, safety planning and clear communication at discharge to mitigate distress and risk after ED presentation (Virk et al. 2022).

Rural ED environments in this study were described as overstimulating and poorly suited to psychological crisis, in addition to concerns about limited mental health staffing and scarce to non‐existing youth inpatient options. Participants advocated for dedicated, calming mental health spaces within ED and for stronger community‐based alternatives to ED where appropriate. These findings are congruent with recommendations for ED environmental design and alternative and integrated crisis pathways (Liddicoat 2019; Hansen et al. 2025; Hudson and Randall 2026). Participants also highlighted the “all roads lead to ED” dynamic, driven by gaps in community options and follow‐up. Investing in continuity of mental health care post‐discharge via comprehensive multi‐component approaches is imperative as it reduces recurrence of suicidal behaviours and subsequent ED presentations.

Process barriers were also evident. Participants reported lengthy waiting times, often in public waiting areas, and noted that consumers sometimes left prior to completion their care. They also observed rigid sequencing of medical clearance before a mental health assessment could be undertaken. These procedural constraints may increase distress, require consumers to repeatedly recount personal and traumatic experiences, and create ethical tensions for staff attempting to balance clinical priorities with compassionate care. The findings reinforce recommendations that EDs adopt more integrated models of concurrent medical and mental health assessment, reduce retraumatisation associated with repeated histories, and ensure timely follow‐up, particularly during the high‐risk period following self‐harm or ED discharge. Staff also identified the potential value of observational tools to support ongoing monitoring of consumers' mental state, including cognition, alertness and emotional status.

Finally, some staff described limited preparation for suicide‐related presentations, describing on‐the‐job learning and variable modelling of best practice, relying heavily on on‐the‐job learning and informal modelling of practice. Targeted suicide‐specific education for ED clinicians, combined with reflective practice and opportunities for debriefing, may strengthen clinicians' confidence in asking direct questions about suicide and promote greater consistency in care delivery. These findings are consistent with prior research exploring ED clinicians' experiences (McCarthy et al. 2024) and with contemporary guideline recommendations for workforce capability building (Hill et al. 2023).

Taken together, these findings highlight the need for system‐level responses that strengthen collaboration between emergency and mental health clinicians, improve the suitability of ED environments for psychological crisis care and enhance workforce capability in responding to suicidal distress. These implications are explored further in the following recommendations.

### 4.1 Recommendations and Future Directions

Findings from this study point to several opportunities to strengthen responses to suicidal distress within rural EDs, particularly through closer collaboration between emergency and mental health clinicians. Participants highlighted the importance of improving the physical and organisational environment of EDs to better support people experiencing a suicidal crisis. Dedicated, low‐stimulus mental health spaces within EDs were frequently identified as a priority, enabling privacy, de‐escalation and therapeutic engagement (Liddicoat 2019). Where possible, such spaces should be staffed or co‐staffed by emergency and mental health nurses to facilitate timely assessment, shared decision‐making, and continuity of care. These recommendations align with system reform priorities identified in the Royal Commission into Victoria's Mental Health System (Victorian State Government 2021).

Strengthening workforce capability was also identified as an important direction for practice. Suicide‐specific education for ED nurses, delivered in partnership with mental health clinicians, may enhance clinicians' confidence in asking direct questions about suicide, responding to distress and understanding the scope of their role in mental health care. In addition, expanding roles for nurses trained in both emergency and mental health nursing may help bridge disciplinary perspectives and support communication, clinical leadership and education within ED teams. Previous rural emergency department research has similarly highlighted the value of such integrated roles in supporting collaborative care (Veresova et al. 2024).

Improving communication between emergency and mental health clinicians may also be supported through the use of brief observational or screening tools that allow staff to track changes in consumers' mental state, including alertness, cognition and emotional presentation. While some participants reported familiarity with mental health assessment tools, their length and complexity limited feasibility in busy ED settings. Adaptation or co‐design of shorter observational tools, developed in collaboration with mental health clinicians, may support more rapid and repeated assessment while maintaining clinical utility.

More broadly, service pathways should strengthen person‐centred engagement and incorporate structured opportunities for family or carer involvement, where appropriate and with youth consent. Streamlining care processes by enabling concurrent medical and mental health assessments, rather than strictly sequential models, may reduce delays, minimise repeated recounting of distressing histories and improve consumer experiences of care. Given the heightened risk period following ED discharge, strengthening continuity of care through timely follow‐up (e.g., within 24–72 h) and clear handover to community mental health services is critical. Particular attention is required in rural areas where community resources and youth‐specific pathways remain limited.

Future research should further examine models of integrated emergency and mental health care in rural settings, including the effectiveness of co‐staffed mental health spaces, collaborative training approaches and brief observational tools for mental health assessment in ED environments. Research that incorporates the perspectives of consumers, families, and multidisciplinary clinicians will be important in informing service design and policy development aimed at improving care for people presenting with suicidal distre.

## 5 Limitations

Several limitations should be considered when interpreting these findings. First, a purposive convenience sampling approach was employed, whereby all staff who expressed interest in participating were enrolled. While this facilitated recruitment in a context where access to participants can be challenging, it introduces the possibility of self‐selection bias. Staff who volunteered to participate may have held stronger views, positive or negative, about mental health care in their ED, which may not be representative of the broader workforce across rural emergency settings.

Second, the study relied on self‐report data collected through individual interviews and focus groups conducted in a specific organisational and geographic context. Participants' accounts reflect their subjective perceptions and experiences at a particular point in time, within two rural Victorian hospitals. The findings may not be transferable to rural EDs with different staffing structures, service configurations or cultural contexts. That said, rich contextual description has been provided to support readers in making informed judgements about the applicability of these findings to other settings.

Third, limitations related to data collection methods are worth acknowledging. Participants were able to choose between an individual interview and a focus group. While this flexibility supported recruitment and participant comfort, it introduced variability in the data collection context. Individual interviews allowed for in‐depth exploration of personal experiences, whereas focus groups generated collective accounts that may have been shaped by group dynamics, including the potential for social desirability, whereby participants present themselves or their workplace in a more favourable light in front of peers.

Finally, as with all qualitative research using reflexive thematic analysis, the analytic process is inherently interpretive and subjective. Themes were constructed through the active engagement of the research team with the data, informed by their clinical and research backgrounds in mental health and emergency care. While this positionality enriched the interpretive process, it also represents a potential source of bias.

## 6 Conclusion

This study explored rural ED staff experiences of caring for people presenting with suicidal thoughts and behaviours and identified the key barriers and facilitators shaping this care. Staff demonstrated a strong commitment to compassionate, person‐centred support, yet faced challenges related to resource limitations, environmental constraints, care pathway delays and gaps in suicide‐specific training. Addressing these structural and procedural barriers through improved ED environments, streamlined assessment processes, strengthened community pathways and targeted workforce development is essential to increase the safety, timeliness and quality of care for people in suicidal distress in rural settings.

## 7 Relevance to Clinical Practice

The findings highlight the need for strengthened integration between ED and mental health services in rural hospital settings. Participants described limited access to specialist mental health support, long waiting times for assessment and environments that were not suitable to supporting people in suicidal distress. These challenges point to the importance of dedicated mental health liaison roles within EDs, improved access to specialist assessment and the availability of designated mental health spaces or beds to support safe and therapeutic care. In addition, targeted suicide‐specific education for ED clinicians and clearer care pathways linking EDs with community‐based crisis and follow‐up services are needed. Strengthening collaboration between ED and mental health services, alongside improved resourcing and training, may help reduce delays in care, support clinicians in managing complex presentations and improve safety and outcomes for people presenting in suicidal crisis in rural communities.

## Funding

This work was supported by the Murray Primary Health Network.

## Ethics Statement

Full ethics approval was obtained from La Trobe University Human Ethics Committee (Approval number HEC25032).

## Conflicts of Interest

The authors declare no conflicts of interest.

## Data Availability

Research data are not shared.
